# Bacterial Load Comparison of the Three Main Lineages of *Mycobacterium tuberculosis* Complex in West Africa

**DOI:** 10.3389/fmicb.2021.719531

**Published:** 2021-10-27

**Authors:** Stephen Osei-Wusu, Portia Morgan, Prince Asare, Godfrey Adams, Abdul Basit Musah, Ishaque Mintah Siam, Stephen Henry Gillespie, Wilber Sabiiti, Dorothy Yeboah-Manu

**Affiliations:** ^1^Noguchi Memorial Institute for Medical Research, University of Ghana, Accra, Ghana; ^2^West African Center for Cell Biology of Infectious Pathogens, University of Ghana, Accra, Ghana; ^3^Department of Chest Diseases, Korle-Bu Teaching Hospital, Accra, Ghana; ^4^Division of Infection and Global Health, School of Medicine, University of St Andrews, St Andrews, United Kingdom

**Keywords:** West Africa, *Mycobacterium africanum*, bacterial load, diversity, *Mycobacterium tuberculosis*

## Abstract

Studies have shown an association between bacterial load and virulence; however, not much is known about the diversity in this phenotypic characteristic of *Mycobacterium tuberculosis* complex (MTBC). This study was therefore aimed to determine the differences in bacterial load of the three most prevalent MTBC genotypes (L4, L5, and L6) in West Africa at the time of diagnosis. A total of 170 paired fresh sputum samples were collected; one part in guanidinium thiocyanate (GTC) was used for RNA extraction and tuberculosis molecular bacterial load assay (TB-MBLA), and the other part without GTC was confirmed for TB positivity using GeneXpert MTB/RIF, smear microscopy grading, and culture on Löwenstein–Jensen media slants. The 170 sputum samples comprised 155 new cases, three follow-up cases, and 12 TB negative sputum samples. The time-to-culture positivity (TTP) and degree of culture positivity (DCP) were recorded. All 122 isolates obtained were spoligotyped for lineage (L) classification, but spoligotypes were obtained from 120 isolates. Of the typed isolates, 70.0, 10.8, 10.8, 4.2, 2.5, 0.8, and 0.8% were lineages 4, 5, 6, 2, 3, 1, and *Mycobacterium bovis*, respectively. Further analysis of the three most prevalent lineages showed significantly shorter TTP and higher DCP by L4 compared to L5 and L6, respectively: TTP 20.8, vs. 26.5, and 28.2 days; *p*-value = 0.005 and DCP 1.27, vs. 0.81 and 0.29, *p* < 0.001. The average TB-MBLA measured bacterial load of L4 was 3.82 Log_10_eCFU/ml which was not significantly different from 3.81 and 3.80 Log_10_eCFU/ml of L5 and L6, respectively, *p* = 0.84. Degrees of smear microscopy L4 = 1.20, L5 = 1.20, and L6 = 0.92 and GeneXpert Cq values L4 = 17.08, L5 = 18.37, and L6 = 17.59 showed no significant difference between the lineages, *p* = 0.72 and *p* = 0.48, respectively. Retrospective analysis of a larger sample confirmed the difference in TTP, *p* < 0.001. In conclusion, the observed shorter TTP and high DCP of L4 could signify high growth rate in culture that is independent of total bacterial load at diagnosis.

## Introduction

Tuberculosis (TB) remains a global health challenge and the leading cause of death from a single infectious pathogen (WHO, 2020). In 2019, it was estimated that about 10 million individuals fell ill from TB with an estimated 1.4 million deaths (WHO, 2020). Human TB is mostly caused by *Mycobacterium tuberculosis* sensu stricto (Mtb) and *M. africanum* (Maf) which are members of the *M. tuberculosis* complex (MTBC) (Gagneux et al., 2006). Members of the MTBC appear genetically monomorphic yet host specific but occasionally cross-infect other hosts (Gagneux et al., 2006; Jagielski et al., 2016). A further classification of the MTBC clusters the human-adapted species into lineages with specific geographical distribution. The Mtb is made up of six lineages (L1-4, L7, and L8) which are generally widely distributed globally while Maf comprising lineages 5 and 6 and the newly identified lineage 9 are mainly restricted to West Africa for reasons not clearly understood (Filliol et al., 2006; Coll et al., 2014; Nebenzahl-Guimaraes et al., 2016; Ngabonziza et al., 2020; Coscolla et al., 2021).

*Mycobacterium africanum* has been reported to cause up to about 50% of human TB in some West African countries as well as four other lineages (lineages 1, 2, 3, and 4) causing TB infection in the subregion (de Jong et al., 2010). In total, six different lineages have been reported to cause TB in West Africa, making the subregion one of the geographical areas with the highest diversity of the pathogen (de Jong et al., 2010; Yeboah-Manu et al., 2016). However, L4, L5, and L6 are the most prevalent genotypes in West Africa (de Jong et al., 2010; Asante-Poku et al., 2016; Yeboah-Manu et al., 2016).

Infection with MTBC does not necessarily imply having the TB disease. There are three main possible outcomes upon entry into the lungs: (1) the innate immunity may be able to clear the pathogen leading to no disease, (2) the pathogen may be contained in the lungs to form granuloma by attracting other immune effector cells to surround the activated macrophages to reduce tissue damage and proliferation of the bacteria, thereby leading to establishment of latent TB infection, and (3) the immune system may not be able to keep the pathogen under check, allowing an active replication of the bacilli and hence leading to active TB disease (Handzel, 2013; de Martino et al., 2014, 2019). These outcomes of TB infections can be attributed to the host immune response, the environmental conditions, and the bacterial factors such as its virulence (Handzel, 2013).

One of the key virulence factors of bacteria is its ability to grow fast to large numbers to frustrate the host immunity; thus, the bacterial load at diagnosis of TB could be used as a marker for virulence (Forrellad et al., 2013; Tram et al., 2018). More virulent lineages of MTBC are able to replicate better under the harsh conditions of the host macrophages, leading to high bacterial load required for subsequent transmission events (Tram et al., 2018). Although studies have shown that there is an association between bacterial load and virulence, not much is known about the diversity in this phenotypic characteristic of MTBC (Malik and Godfrey-Faussett, 2005; Tram et al., 2018). Different strains of the bacilli may express different levels of bacterial load in sputum based on the variation in their virulence, which may lead to different disease outcomes. Our aim therefore was to determine the differences in bacterial load of the three most prevalent MTBC genotypes in West Africa (L4, L5, and L6) at the time of diagnosis.

## Materials and Methods

### Ethical Consideration

Ethical clearance for the study was obtained from the Institutional Review Boards (IRB) of Noguchi Memorial Institute for Medical Research (NMIMR), University of Ghana (federal-wide assurance number: FWA00001824) and the Korle-Bu Teaching Hospital (KBTH). The ethical clearance documentation included informed consent of participants to be enrolled voluntarily. For participants below 18 years, child assent was sought from the participants and consent from their parents or guardians. After the consent was obtained, a questionnaire was administered to the participants to capture their demographic data.

### Study Design

Patients showing clinical symptoms of active pulmonary TB (PTB) and sputum positive for GeneXpert MTB/RIF were prospectively recruited into the study. Samples were collected between November 2018 and October 2020 from three health facilities in the Greater Accra region of Ghana: Korle-Bu Teaching Hospital, University of Ghana hospital, and “37 Military” Hospital. This study sought to recruit new TB cases of all ages who reported to the above listed health facilities; however, a few follow-up cases were included.

### Sample Collection and Bacterial Cultivation

Two tubes of spontaneously expectorated sputum samples were collected from each PTB patient before the start of anti-TB therapy. To preserve the RNA, samples in the first tubes were treated with guanidine thiocyanate (GTC) solution within 15 min of sputum production and stored at −80°C. The second tubes with sputa were transported to the laboratory for bacteria cultivation. The samples were decontaminated by 5% oxalic acid, as previously described (Yeboah-Manu et al., 2004) and inoculated on four Löwenstein–Jensen (LJ) media slants; two slants supplemented with glycerol and the other two supplemented with pyruvate. The inoculated slants were incubated at 37°C and observed every day for the first week for possible contamination and subsequently observed for macroscopic growth. The time to culture positivity (TTP) was recorded as the number of days it took for the first mycobacterial-like growth to be observed and the DCP according to the Mycobacteriology Laboratory Manual (Stinson et al., 2014) was also recorded. The TTP and DCP were used to assess the relationship of lineage and growth rate and implications for bacterial load.

**FIGURE 1:**
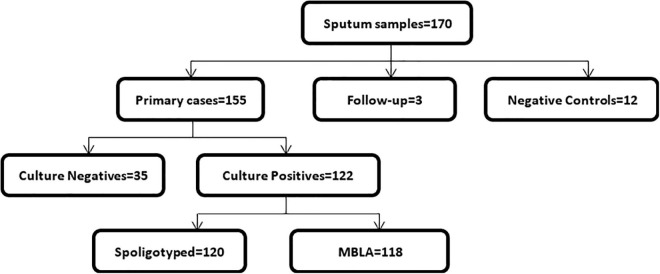
The study workflow of sputum sample collection.

In total, we collected 170 sputum samples comprising 155 new cases, three follow-up cases, and 12 TB negative sputum samples as controls (Figure 1). Majority of the cases (*n* = 61; 39.9%) were within the age group of 31–45 years, followed by 41 (26.8%) in the 15–30-year group, 37 (24.2%) in the 46–60-year group, 13 (8.5%) above 60 years, and 1 (0.6%) case below the age of 15. Most of the TB cases 118/158 (75%) were males, and the remaining 25% were females.

The 12 negative control participants comprised of eight (66.7%) males and four (33.3%) females, and they were in the age categories of 15–30 (*n* = 5, 41.7%), 31–45 (*n* = 4, 33.3%), and 45–60 (*n* = 3, 25.0%).

### Smear Microscopy

Smears were made from the sediments obtained after decontamination on microscope slides. The slides were stained with Ziehl–Neelsen staining technique and microscopically examined for the presence of acid-fast bacilli. The bacterial load at the time of diagnosis was estimated as the number of acid-fast bacilli which was recorded as the degree of positivity according to the WHO standard (WHO et al., 2001).

### *Mycobacterium tuberculosis* Complex Lineage Identification

All obtained mycobacterial isolates were first confirmed as members of the MTBC by amplification of the Insertion Sequence *6110* (IS*6110*). Mycobacterial isolates confirmed as MTBC were then genotyped by spacer oligotyping (spoligotyping) by amplification of the direct repeat (DR) region followed by hybridization onto a film. Binary data obtained from either the presence or absence of spacers in the DR region were analyzed in the MIRU-VNTR*plus* database to determine the infecting lineage/sub-lineage (Kamerbeek et al., 1997).

### *GeneXpert MTB/RIF* Assay

The bacterial load in all the sputum samples obtained was quantified using Xpert MTB/RIF Ultra (Cepheid, Sunnyvale, CA, United States), an automated nucleic acid amplification (NAA) test. The quantification cycle (Cq) values from the real-time PCR assay which targets the *rpoB* gene with five probes were used for the quantification of mycobacterial DNA. This method uses a disposable cartridge with the GeneXpert (GX) instrument as per the manufacturer’s instructions (WHO, 2013a). Briefly, fresh sputum samples collected from TB suspected patients were mixed with GX reagent (2 × the sample volume) and incubated for 15 min at room temperature with intermittent shaking. An aliquot of 2 ml of each diluted sample was transferred into the cartridge and subsequently run in the GX instrument. Results were reported as the average quantification cycle (Cq) values of five probes, which was used to estimate bacterial load. Data generated were analyzed with the GX software version 4.3 where the mean Cq values were categorized as high (Cq < 16), medium (Cq 16–22), low (Cq 22–28), and very low (Cq > 28) (WHO, 2013a, b).

### Tuberculosis-Molecular Bacterial Load Assay

The TB molecular bacterial load assay (TB-MBLA) is a reverse transcriptase real-time quantitative PCR (RT-qPCR) method that detects and quantifies viable MTBC in sputum by determining the amount of MTBC 16S ribosomal RNA (16S rRNA). It is a two-step process consisting of a) extraction of total RNA and b) RT-qPCR (Sabiiti et al., 2017, 2020b).

#### Extraction of Total RNA

Spontaneously expectorated sputum samples collected into GTC solution were used for RNA extraction. An aliquot of 1 ml of the homogenized sputum was collected into a new vial, and total RNA was extracted using FastRNA Pro Blue Kit (MP Biomedicals LLC, Illkirch-Graffenstaden, France) according to the manufacturer’s instruction. Briefly, 100 μl of internal control (IC) suspension was added directly into each sample followed by cell lysis. Phase separation was obtained by adding the lysed cells to 300 μl of chloroform. The aqueous phase was pipetted out into a new 2-ml vial followed by the addition of 500 μl of 100% ice-cold ethanol and incubated at −20°C overnight. The extracted RNA was washed with 70% ice-cold ethanol and dried at 50°C in a heating block. The extract was then treated with Turbo DNase using the Turbo DNA-free Kit (Ambion AM1907, Thermo Fisher Scientific, Vilnius, Lithuania) to remove genomic DNA following the manufacturer’s instruction.

#### Reverse Transcriptase–Quantitative PCR

Specific primers and probes for the MTBC 16S ribosomal RNA were added to the master mix (QuantiTect Multiplex RT-PCR NR Kit (QT) (Qiagen Inc., Hilden, Germany) (Sabiiti et al., 2020b). All RNA samples were analyzed in duplicates: as diluted (1 in 10 dilution) and neat (without dilution). The Cq values for the duplicate standard curves were constructed with known RNA concentrations for MTBC and the extraction control (Vitalbacteria St Andrews Applied Research Ltd., United Kingdom) which served as reference for translating Cq values into bacterial load measured as estimated colony forming unit per milliliter (eCFU/mL).

### Data Analysis

Statistical analyses to determine the differences between the three lineages were carried out using analysis of variance (ANOVA) in RStudio Version 1.2.5033 (RStudio, Inc., Boston, MA, United States). Further specific differences were determined with *ad hoc* test and Tukey test. The significance level for all statistical analyses was set at a p-value less than 0.05 at a confidence level of 95%. Figures were developed using RStudio, and data for the three lineages were represented by their respective standard color codes: red = L4, brown = L5, and green = L6.

## Results

### Sputum Samples and Mycobacterial Isolates

Out of the 155 new cases, 122 were culture positive. Therefore, the culture positivity rate was calculated as 78.7%. We were able to genotype 120 of the 122 culture-positive isolates by spoligotyping, of which TB-MBLA was carried out for 118. GeneXpert MTB/RIF Cq values were obtained for all the samples received. All the negative control sputum samples and the follow-up cases were negative for culture on L-J media slants.

### *Mycobacterium tuberculosis* Complex Lineages and Sub-Lineages

All six lineages prevalent in West Africa were observed in this study but at different frequencies. The highest prevalent lineage was L4 with 84 (70.0%) isolates (Table 1). The sub-lineages under L4 were Cameroon (53, 44.2%), Ghana (17, 14.2%), Haarlem (7, 5.8%), Uganda1 (5, 4.2%), X (1, 0.8%), and X3 (1, 0.8%). There were 13 (10.8%) L5 and 13 (10.8%) L6 isolates as well as 5 (4.2%) L2 isolates all belonging to the Beijing sub-lineage. There were three (2.5%) L3 isolates all belonging to the Delhi/CAS sub-lineage. In addition, we observed one (0.8%) L1 isolate of the EAI sub-lineage and one (0.8%) *Mycobacterium bovis* isolate. Comparison of results was made only between the three main prevalent lineages in West Africa and Ghana, that is, L4, L5, and L6.

**TABLE 1:** Frequency of lineages and sub-lineages.

**Lineage**	**Sub-lineage**	**Total number (%)**
	Cameroon	53 (44.2)
	Ghana	17 (14.2)
L4	Haarlem	7 (5.8)
	Uganda1	5 (4.2)
	X	1 (0.8)
	X3	1 (0.8)
L5	L5	13 (10.8)
L6	L6	13 (10.8)
L2	Beijing	5 (4.2)
L3	Delhi/CAS	3 (2.5)
L1	EAI	1 (0.8)
*M. bovis*	*bovis*	1 (0.8)
Total		120

### Mycobacterial Load at Diagnosis

**FIGURE 2:**
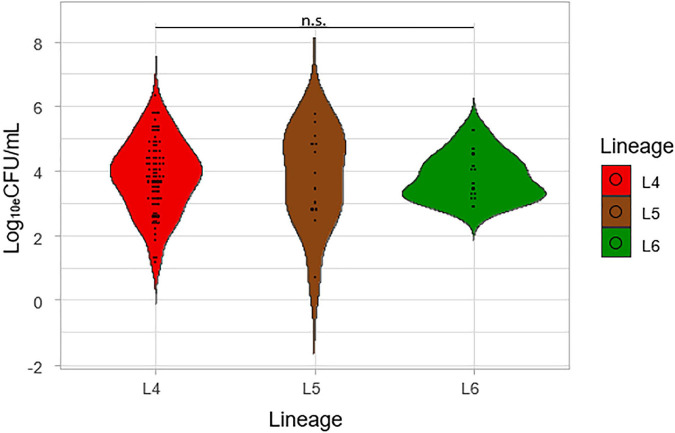
Comparison of the colony-forming unit per mL (Log_10e_CFU/mL) of the lineages. Log_10e_CFU/mL values obtained from MBLA were plotted against the three lineages.

Three microbiological methods were used to determine the bacterial load of the prevalent genotypes of MTBC in West Africa. The log_10_eCFU/mL values obtained from TB-MBLA were plotted against the three lineages (Figure 2). Although the average log_10_eCFU/mL of L4 was the highest (3.82), followed by L5 (3.81) and L6 (3.80) respectively, there was no observed significant difference, ANOVA *p* = 0.84.

**FIGURE 3:**
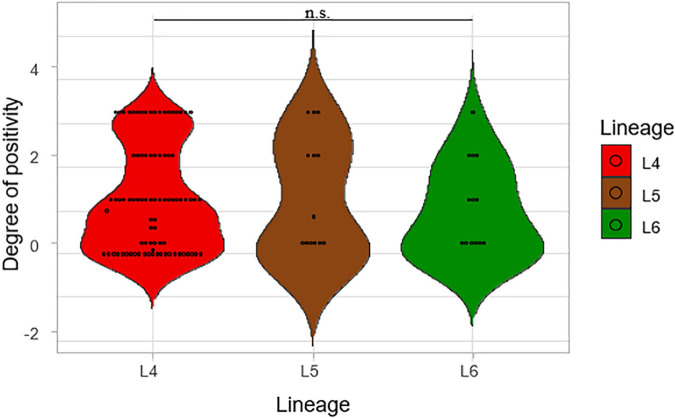
Comparison of smear microscopy results of the lineages. The degree of positivity from the smear microscopy was compared for the three lineages.

**FIGURE 4:**
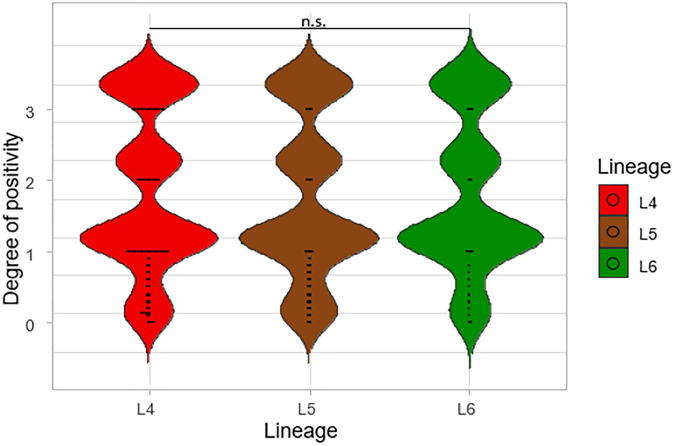
A large-scale comparison of the smear microscopy results of the lineages. The degrees of positivity of the lineages were compared using a total number of 2,043 isolates from a previous study (L4 = 1,563, L5 = 289, and L6 = 191).

Degrees of smear microscopy for the three lineages were compared revealing no significant difference across the three lineages, ANOVA *p* = 0.72 (Figure 3). The average degrees of positivity for both L4 and L5 were the same (1.20) with 0.92 for L6. We further compared the smear microscopy results of retrospective data with a larger sample size of 2,043 isolates (Asare et al., 2018) (L4 = 1,563, L5 = 289, L6 = 191) (Figure 4). Lineage 4 showed the highest average degree of positivity of 1.57 followed by L6 (1.52) and then L5 (1.48), and still no significant difference was observed ANOVA, *p* = 0.38.

**FIGURE 5:**
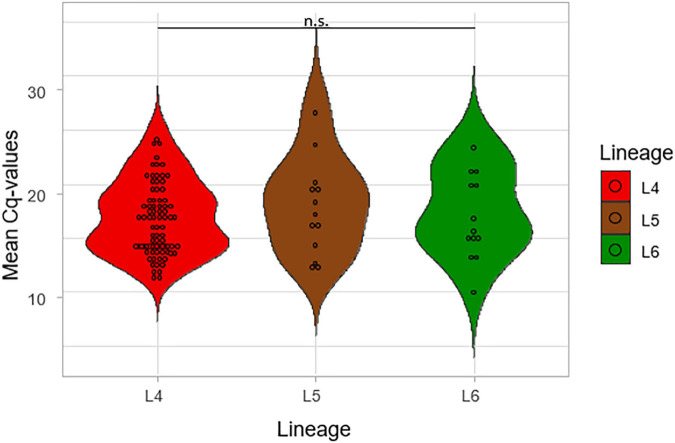
Comparison of the mean Cq values of the lineages using Gene Xpert. The mean of the Cq values of the five probes from the GeneXpert machine was calculated and plotted against the three lineages.

The mean of the Cq values for the five probes from the Xpert MTB/RIF Ultra was compared among the three lineages, as shown in Figure 5. The lowest average of the mean Cq-value was recorded for L4 (17.08) followed by L6 (17.59) and L5 (18.37), respectively. The lower the Cq, the higher the bacterial load, but the difference was not significant across the three lineages (*p* = 0.48), which is consistent with TB-MBLA measured bacterial load.

### The Relationship of Lineage and Growth Rate

**FIGURE 6:**
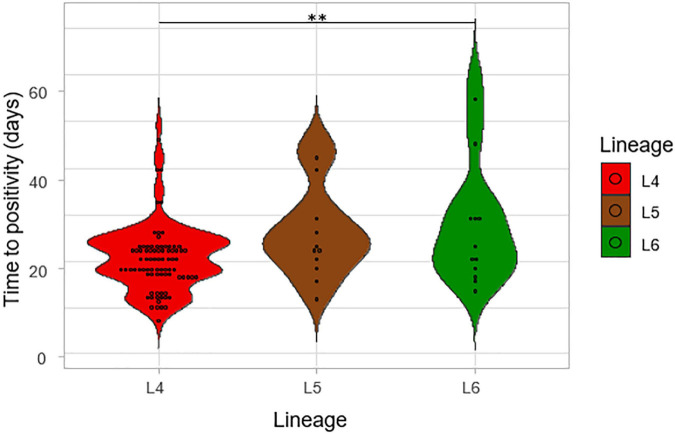
The time to positivity (TTP) on L-J media slants for the lineages. The number of days for the first distinct colony to appear on the media slant was recorded as the time to positivity for the lineages.

**FIGURE 7:**
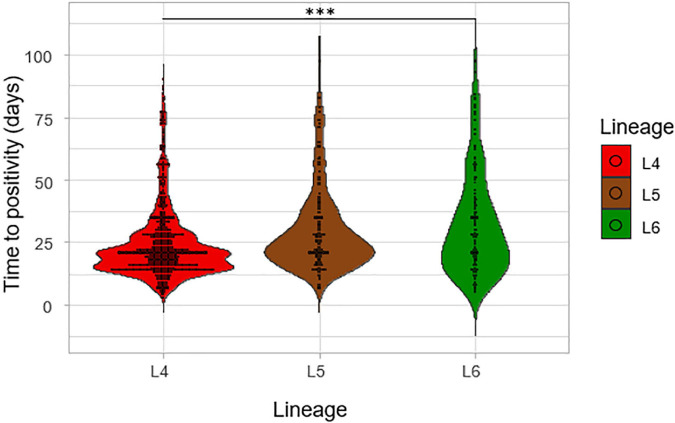
Time to positivity on L-J media slants considering a larger sample size. The TTP of the three lineages was assessed for a previous study with a total of 2,219 isolates (L4 = 1,712, L5 = 306, and L6 = 201).

The impact of lineage on growth rate was assessed by using the time to positivity (TTP) and degree of culture positivity (DCP) on L-J media slants for the isolates. The number of days for the first distinct colony to appear on the L-J media slant was recorded as the TTP for the three lineages (Figure 6). There was a significant difference in the TTP among the lineages, ANOVA *p* = 0.005. Lineage 4 showed the lowest average TTP (20.8 days) followed by L5 (26.5 days) and then L6 (28.2 days). The difference seems to have been driven by the difference between L4 and L6 (*p* = 0.01) because there was no significant difference between L5-L6 (*p*-value = 0.87). Since there was a propensity for a significant difference between the TTP of L4 and that of L5 (*p*-value = 0.09), we assessed this in a large sample size by retrospectively analyzing data generated from a previous study with a larger sample size (Asare et al., 2018) with a total of 2,219 isolates (L4 = 1,712, L5 = 306, L6 = 201), as shown in Figure 7. Again, L4 showed the lowest average TTP (23.7 days) followed by L5 (29.4 days) and then L6 (31.9 days). There was also a significant difference between the TTP of the lineages (*p*-value < 0.001). In these data, there was significant difference between L4-L6 (*p* < 0.001) and L4-L5 (*p* < 0.001). Again, there was still no significant difference between L5 and L6 (*p* = 0.13).

**FIGURE 8:**
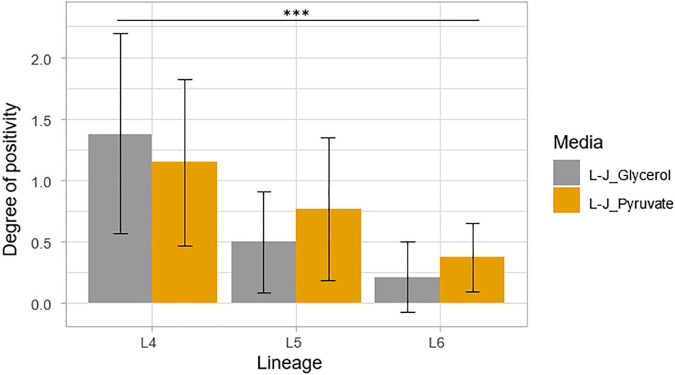
Degree of culture positivity on L-J slants supplemented with either glycerol or pyruvate. Positivity was recorded at the same time as the TTP. Error bars were generated from the standard deviation (SD).

Assessment of the degree of culture positivity also showed that L4 had the highest average degree of culture positivity of 1.27 with an average DCP of 1.38 on L-J supplemented with glycerol (L-J_Glycerol) and 1.15 on L-J supplemented with pyruvate (L-J_Pyruvate), as shown in Figure 8. Lineage 5 followed with an average DCP of 0.81 (average DCP on L-J_Glycerol = 0.69; L-J_Pyruvate = 0.94) and L6 had the least average DCP of 0.29 (average DCP on L-J_Glycerol = 0.21; L-J_Pyruvate = 0.37). There was a significant difference between the three lineages, *p* < 0.001, specifically between L4 and L5 (*p* < 0.001) and between L4 and L6 (*p* < 0.001). However, there was no statistical significance difference between L5 and L6 (*p* = 0.20).

## Discussion

The virulence of MTBC can be evaluated by its (1) ability to survive in the host, (2) ability to multiply in the midst of the harsh host environment, and (3) ability to be transmitted successfully to new host for its propagation (Coscolla and Gagneux, 2014; Echeverria-Valencia et al., 2018; Ly and Liu, 2020). The MTBC is able to exploit the host machinery to transmit to other individuals through the evolution of their specific adaptations to the host immunity (Mehrotra et al., 2014; Brites and Gagneux, 2015). The higher the bacterial load of an index TB patient, the higher the chance of successful transmission to another person (Turner and Bothamley, 2014; Turner et al., 2017; Migliori et al., 2018). Therefore, high bacterial load is essential to ensuring continuous propagation of the MTBC and hence considered a very important virulence factor (Rouillon et al., 1976; Salinas et al., 2007; Brites and Gagneux, 2015). This property can be exploited to investigate the differences in virulence for the different lineages of MTBC. We explored and compared bacterial load of the three dominant MTBC lineages in West Africa (L4, L5, and L6) using TB-MBLA, sputum-smear microscopy, Xpert MTB/RIF Ultra, time-to-culture positivity, and degree of culture positivity.

We found that L4 had the lowest TTP and shows the fastest growth on L-J media slants followed by L5 and then L6. Also, L4 showed the highest degree of culture positivity compared to L5 and L6. This suggests that either L4 replicates at a relatively faster rate or the sputum samples containing L4 had higher bacterial load than the Maf lineages (L5 and L6). To verify this, we compared the TB-MBLA results for the lineages which confirmed L4 with the highest bacterial load, but this was not significantly different from L5 and L6. The insignificant difference in bacterial loads of the three lineages is an indication that the growth rate in culture cannot be fully explained by the underlying bacterial load. Studies have shown the existence of dormant bacilli that cannot grow in culture and may explain why the growth rate does not correlate with bacterial load particularly measured by assay like TB-MBLA that detects both dormant and actively growing bacilli (Mukamolova et al., 2010; Dusthackeer et al., 2019). The trend of higher bacterial load and shorter time-to-culture positivity demonstrated by L4 is consistent with the reported inverse relationship between TTP and TB-MBLA measured bacterial load (Sabiiti et al., 2020b). Since TB bacillary load has been shown as an early marker of disease severity, future studies should investigate the relationship between L4 with disease severity and treatment outcomes (Sabiiti et al., 2020a).

The assessment of the sputum-smear microscopy gradings of the three MTBC lineages showed no significant difference in the bacterial load. The low sensitivity of smear microscopy of 20–80% in TB prevalent regions could account for its potential inaccurate estimation of bacterial loads (WHO, 2009; Chadha et al., 2019). Therefore, to verify that L4 either has the highest bacterial load or as the fastest replication compared to L5 and L6, we compared Xpert MTB/RIF Cq values among the three lineages. The lower the mean Xpert MTB/RIF Cq value, the higher the bacterial load. Although L4 had the lowest mean Cq values followed by L6 and L5, respectively, the difference was not statistically significant. Tram et al. (2018) were able to show an association between MTBC virulence and bacterial load using a linear trend test. Although they showed that increased MTBC virulence was associated with increased bacterial load using the East Asian/Beijing lineage (Tram et al., 2018), they did not compare the bacterial load of different lineages as done in this study.

Overall, there was no significant difference in the bacterial load for the three lineages using smear microscopy, Xpert MTB/RIF, and TB-MBLA. Our findings corroborate a previous study where they showed a similar transmission for both Mtb and Maf but different rates of progression to disease (de Jong et al., 2008). Our study showed that all three lineages had similar bacterial loads at the point of diagnosis, but there was diversity in the TTP and DCP.

Moreover, TB patients irrespective of the infection genotype may have high variation in the time between exposure and the time of reporting to the hospital. This can potentially affect the time each genotype/lineage of the MTBC has to replicate within each affected TB patient; hence, there is no correlation between replication rate and bacterial load at the time of diagnosis. Additionally, a major challenge to the fight against TB in Ghana and Africa is stigmatization (Lawn, 2000; Dodor et al., 2008). Due to stigmatization, many individuals with signs and symptoms of TB are reluctant to report to the hospital for diagnosis (Dodor et al., 2008; Amo-Adjei, 2016). This leads to delay in reporting of cases, consequently leading to high bacterial burden before treatment irrespective of the infecting lineage of the MTBC.

## Conclusion

In conclusion, our data show that L4 grows faster than L5 and L6 irrespective of the pretreatment bacterial load. This can possibly be attributed to the intrinsic properties of L4 such as higher proportion of actively growing bacilli that can easily be recovered in culture. We therefore recommend a larger study that will recruit more participants infected with the different MTBC lineages who can be monitored to determine the difference in their rate of sputum bacterial load clearance and treatment outcomes using the TB-MBLA in comparison to the current standard-of-care tests.

## Data Availability Statement

The original contributions presented in the study are included in the article/supplementary material, further inquiries can be directed to the corresponding author/s.

## Ethics Statement

The studies involving human participants were reviewed and approved by Institutional Review Boards (IRB) of Noguchi Memorial Institute for Medical Research (NMIMR), University of Ghana (Federal wide assurance number: FWA00001824) and the Korle-Bu Teaching Hospital (KBTH). Written informed consent to participate in this study was provided by the participants’ legal guardian/next of kin.

## Author Contributions

DY-M and SO-W: conceptualization and writing—original draft. SO-W, AM, IS, PM, GA, and PA: data curation. SO-W and WS: formal analysis. DY-M, WS, and SG: funding acquisition and resources. SO-W, DY-M, PM, PA, AM, IS, and GA: investigation. SO-W, DY-M, PA, WS, and SG: methodology. DY-M: project administration. DY-M and WS: supervision. SO-W, DY-M, WS, PA, and PM: writing—review and editing. All authors contributed to the article and approved the submitted version.

## Conflict of Interest

The authors declare that the research was conducted in the absence of any commercial or financial relationships that could be construed as a potential conflict of interest.

## Publisher’s Note

All claims expressed in this article are solely those of the authors and do not necessarily represent those of their affiliated organizations, or those of the publisher, the editors and the reviewers. Any product that may be evaluated in this article, or claim that may be made by its manufacturer, is not guaranteed or endorsed by the publisher.
